# Successful Recanalization by Intravenous Thrombolysis in a Patient With Calcified Cerebral Emboli With Major Vessel Occlusion: A Case Report

**DOI:** 10.7759/cureus.52593

**Published:** 2024-01-19

**Authors:** Hajime Ikenouchi, Takuya Saito, Shota Igasaki, Yuichi Kawabata, Yukako Yazawa

**Affiliations:** 1 Neurology, Sendai City Hospital, Sendai, JPN; 2 Cerebrovascular Medicine, Kohnan Hospital, Sendai, JPN

**Keywords:** mobile calcified emboli, acute ischemic stroke, major vessel occlusion, intravenous thrombolysis, calcified cerebral emboli

## Abstract

A 69-year-old man, with a history of left superficial temporal artery-middle cerebral artery bypass due to cerebral infarction by left internal carotid artery occlusion, was hospitalized with acute right hemispatial neglect and left hemiparesis. Diffusion-weighted imaging showed a high-intensity lesion in the right insular cortex. Although there seemed to be no arterial occlusion in magnetic resonance angiography (MRA), non-contrast computed tomography (CT) on admission showed calcification in the right Sylvian fissure. As hyperacute ischemic stroke within 4.5 hours after onset, we used an intravenous recombinant tissue plasminogen activator, and his symptoms improved. Follow-up MRA revealed recanalization of the right M2 branches with distal migration of calcification. Although calcification was identified on non-contrast CT in the initial assessment, the diagnosis of middle cerebral artery occlusion was missed. Therefore, arterial occlusion should be considered when calcification is observed in the brain sulcus. This case also illustrated that intravenous thrombolysis may be effective even in calcified cerebral emboli with major vessel occlusion.

## Introduction

Calcified cerebral emboli (CCE) is a type of cerebral embolism caused by intracranial calcification and a rare cause of cerebral infarction [1]. CCE is often misdiagnosed or overlooked in initial assessment [2]. Although reperfusion therapy, including intravenous thrombolysis (IVT) or endovascular therapy (EVT), could occasionally be used to treat CCE, the efficacy of reperfusion therapy for CCE is controversial [3-5]. This report presents the case of a patient with CCE who missed the diagnosis on the initial assessment but was successfully treated by IVT. This report will inform clinicians of the clues for detecting CCE and the effectiveness of hyperacute treatment in CCE with major vessel occlusion.

## Case presentation

**Figure 1:**
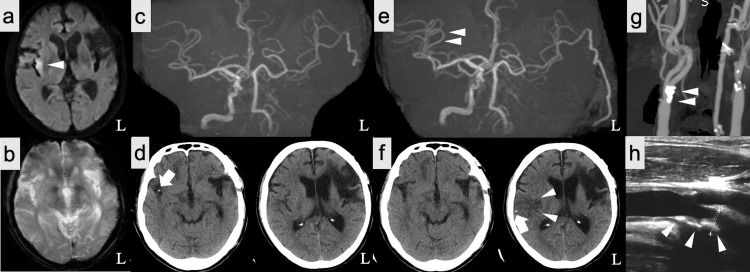
Imaging findings of calcified cerebral emboli in this case a: Diffusion-weighted imaging reveals a high-intensity lesion in the right middle cerebral artery (MCA) territory (arrowhead). b: T2*-weighted gradient echo imaging shows no sign of susceptibility vessel in the right Sylvian fissure. c: Magnetic resonance angiography (MRA) on admission shows no major branch occlusion in the right MCA. d: Axial computed tomography (CT) images reveal a high-density spot suspected to be a calcified embolus in the right Sylvian fissure (arrow). e: Follow-up MRA shows recanalization of the right M2 segment (arrowheads). f: Follow-up CT performed 24 hours after intravenous thrombolysis revealed low-density lesions at the middle MCA territory (arrowheads) and migration of the calcified embolus to the distal MCA (arrow). g: CT angiography shows calcified plaque at the cervical segment of the right internal carotid artery (ICA) (arrowheads). h: Carotid ultrasonography shows calcified plaque at the cervical segment of the right ICA (arrowheads).

A 69-year-old man presented to our hospital within 4.5 hours after left hemiparesis onset. He had a history of left superficial temporal artery-middle cerebral artery (STA-MCA) bypass because of cerebral infarction by left internal carotid artery (ICA) occlusion and took aspirin. He also had a history of hypertension and dyslipidemia and took antihypertensive medications and statin. Neurological examination revealed left hemispatial neglect and moderate left hemiparesis with Medical Research Council (MRC) grade 3 and a National Institutes of Health Stroke Scale score of 13. Brain magnetic resonance imaging (MRI) showed acute cerebral infarction in the right insular cortex in diffusion-weighted imaging (Fig. 1a). T2*-weighted gradient echo imaging showed no susceptibility vessel sign (SVS) in the right Sylvian fissure (Fig. 1b). Moreover, there seemed no occluded branches of the right MCA in the MR angiography (MRA) (Fig. 1c). Brain NCCT revealed calcification in the right Sylvian fissure, which was considered as vessel wall calcification (Fig. 1d). Because of acute ischemic stroke within 4.5 hours after symptom onset, we administered IVT (0.6 mg/kg alteplase). EVT was not performed because there seemed no major vessel occlusion. His symptoms gradually improved after IVT. However, a follow-up MRA on day two revealed recanalization of the right M2 branches (Fig. 1e). NCCT performed revealed low-density lesions in the middle MCA territory and migration of the calcification to the distal MCA (Fig. 1f). Carotid ultrasonography (LOGIQ E8, GE Healthcare, Chicago, IL) and cervical CT angiography revealed stenosis at the cervical segment of the right ICA with ulceration (Fig. 1g, Fig. 1h).

He was diagnosed with CCE due to calcified plaque at the cervical ICA. We added clopidogrel for acute treatment, and dual antiplatelet therapy was continued for two weeks. His symptoms and cerebral infarction did not recur, and he was transferred to a rehabilitation hospital two weeks after his stroke onset.

## Discussion

This report describes a case of right MCA branch occlusion due to CCE. CCE was overlooked in the initial assessment, and its diagnosis was established after successful recanalization by IVT.

CCE is a rare cause of cerebral infarction [1]. It is caused by aortic stenosis, valvular disease, and carotid artery calcification [6-8]. CCE is usually detected with NCCT, which provides a superior diagnosis to MRI [1]. However, CCE can be easily missed on initial evaluation or mistaken for other diseases, such as hemorrhage, vessel wall calcification, or infectious residual [2]. In our case, calcification in the Sylvian fissure was diagnosed as vessel wall calcification at first. However, calcification migrated to the distal MCA following IVT. Therefore, we diagnosed the acute artery occlusion rather than vessel wall calcification. One reason for the difficulty in detecting the occlusion was the history of contralateral STA-MCA bypass, which hindered the comparison of both MCA branches by MRA. The other reason for the missed diagnosis of CCE was the absence of the SVS. Generally, SVS is observed on T2*-weighted gradient echo images and indicates artery occlusion [9]. SVS usually represents a fibrin-rich clot [9], and a calcified embolus, which usually has inorganic calcification, might not show SVS as in our case. The detection of occluded vessels in an acute setting using only MRA might be challenging [10]. Therefore, clinicians should consider acute artery occlusion in case of calcification on NCCT in the brain sulcus in acute ischemic stroke.

Previous reports have highlighted the difficulty of the recanalization of CCE using reperfusion therapy, including IVT or EVT [3-5], possibly because of the embolus stiffness due to calcification. There was a report that mentioned that IVT was inferior to EVT in CCE [11]. In contrast, another report described the successful treatment of CCE with IVT [12], similar to that observed in our case. Although evidence is limited, clinicians should consider reperfusion therapies for CCE because they are the only intervention methods that have the potential to improve symptoms in patients with acute ischemic stroke. Fortunately, recanalization was achieved with IVT alone in this case. However, because of a stiff clot with calcification, it is important to consider a treatment strategy that includes EVT, as well as IVT in cases of CCE with large vessel occlusion.

## Conclusions

This is a case of CCE who missed the diagnosis on the initial assessment. In acute ischemic stroke, artery occlusion due to CCE should be considered when calcification in NCCT is observed in the brain sulcus. In the present case, IVT achieved successful recanalization. Although reperfusion therapy is controversial in CCE patients, clinicians should actively consider reperfusion therapy even if CCE is suspected.
